# Anisakid and Raphidascaridid parasites in *Trachurus trachurus*: infection drivers and possible effects on the host’s condition

**DOI:** 10.1007/s00436-021-07200-0

**Published:** 2021-08-14

**Authors:** Fabio Macchioni, Perla Tedesco, Vanessa Cocca, Andrea Massaro, Paolo Sartor, Alessandro Ligas, Carlo Pretti, Gianfranca Monni, Francesca Cecchi, Monica Caffara

**Affiliations:** 1 Department of Veterinary Sciences, University of Pisa, Pisa, Italy; 2 Department of Veterinary Medical Sciences, Alma Mater Studiorum, University of Bologna, Bologna, Italy; 3 APLYSIA, Livorno, Italy; 4 CIBM, Inter-University Center of Marine Biology and Applied Ecology “G. Bacci”, Livorno, Italy

**Keywords:** *Anisakis* spp., *Hysterothylacium* spp., *Trachurus trachurus*, Infection drivers, Fish condition, Mediterranean Sea

## Abstract

This study investigated the distribution of nematode larvae of Anisakidae and Raphidascarididae (genera *Anisakis* and *Hysterothylacium*) in *Trachurus trachurus* (Linnaeus, 1758) in the Ligurian and central-northern Tyrrhenian Seas. The relationship between the number of parasites and the length and weight parameters of the fish was assessed, and the possible effect of the parasites on the condition factor was evaluated. A total of 190 T*. trachurus* specimens were collected in July 2019. Parasites were found in 70 individuals. A total of 161 visible larvae were collected in the viscera. Morphological analysis revealed the presence of *Anisakis* spp. in 55 fish and *Hysterothylacium* spp. in 15 fish, while 5 fish showed coinfection with both genera. The specimens subjected to PCR (n = 67) showed that 85% of the *Anisakis* larvae analyzed belonged to the species *A. pegreffii*, while the remaining 15% belonged to hybrids of *A. pegreffii*-*A. simplex* (s.s.). A total of 58% (n = 7) of the *Hysterothylacium* larvae analyzed belonged to the species *H. fabri*, while 42% belonged to the species *H. aduncum*. Our results support the hypothesis that infection with these parasites does not affect the condition of the fish host analyzed, and that body size and depth are major drivers in determining infection levels with Anisakid and Raphidascaridid nematodes.

## Introduction

Anisakidosis is a fish-borne zoonosis following ingestion of the third larval stage of nematodes of the family Anisakidae. Within the *Anisakis simplex* Rudolphi, 1809 complex, the species *A. simplex* (s.s.) and *A. pegreffii* Campana-Rouget and Biocca, 1955 (Mattiucci et al. 2014) are recognized as the main causative agents of anisakiasis, a condition related to the consumption of raw, marinated, or undercooked fish filets infected by the third-stage larvae of these parasites. In Mediterranean waters, the species *A. pegreffii* is dominant and is also the main etiological agent of anisakiasis and is distributed in numerous paratenic and definitive hosts (Mattiucci and D’Amelio 2014; Mattiucci et al. 2019).

Species of the genus *Hysterothylacium* Ward and Magath, 1917, formerly belonging to the Anisakidae and currently assigned to the family Raphidascarididae, are common parasites in different marine and freshwater fish species (Bezerra et al. 2020). *H. aduncum* Rudolphi, 1802 and *H. fabri* Rudolphi, 1819 are the most frequently reported species in teleost fish from the Mediterranean region (Roca‐Geronès et al. 2018; Tedesco et al. 2018). Evidence of the direct consequences of *Hysterothylacium* infection on fish health is limited: parasites of this genus are considered only mildly pathogenic for adult fish (Ishikura et al. 1993; Yagi et al. 1996; Valero et al. 2003; Cavallero et al. 2012); however, mortality episodes in larval and juvenile fish have been reported (Bristow 1990; Balbuena et al. 2000). Although generally not listed among fish-borne zoonotic agents, preliminary evidence on the allergenic potential of *Hysterothylacium* species (Fernández-Caldas et al. 1998; Valero et al. 2003) suggests their importance in relation to food safety and human health.

Monitoring the occurrence of *Anisakis* and *Hysterothylacium* in wild fish for human consumption is therefore necessary, particularly regarding selected species (Debenedetti et al. 2019) considered at higher risk of infection. Furthermore, the high parasite load reported in susceptible fish species (Manfredi et al. 2000; Angelucci et al. 2011) highlights the need to investigate the effects of parasites on the host’s condition.

Among Mediterranean fish species at high risk of Anisakids and Raphidascaridids, the Atlantic horse mackerel *Trachurus trachurus* Linnaeus, 1758 (Trachuridae, Carangidae) is a gregarious bentho-pelagic species, widely distributed throughout the Mediterranean Sea including the Black Sea (Bini 1967) and eastern Atlantic from Iceland to Senegal (Abaunza et al. 2008), and supports large fisheries (Abaunza et al. 2003), both as target and by-catch species. This species feeds on small fish and planktonic crustaceans and may become infected by both *Anisakis* and *Hysterothylacium* larvae by consuming euphausiids, which are intermediate hosts of these nematodes (Smith 1983; Adroher et al. 1996).

In the present study, we surveyed the occurrence and distribution of *Anisakis* spp. and *Hysterothylacium* spp. in the Atlantic horse mackerel, *T. trachurus*, caught in the FAO-GFCM Geographic Sub-area 9 (GSA9), Ligurian Sea and central-northern Tyrrhenian Sea, investigating the effect of infection on the host’s condition and the influence of different biological (total length, total weight, sex) and environmental (depth) variables.

## Material and methods

### Study area

**Fig. 1:**
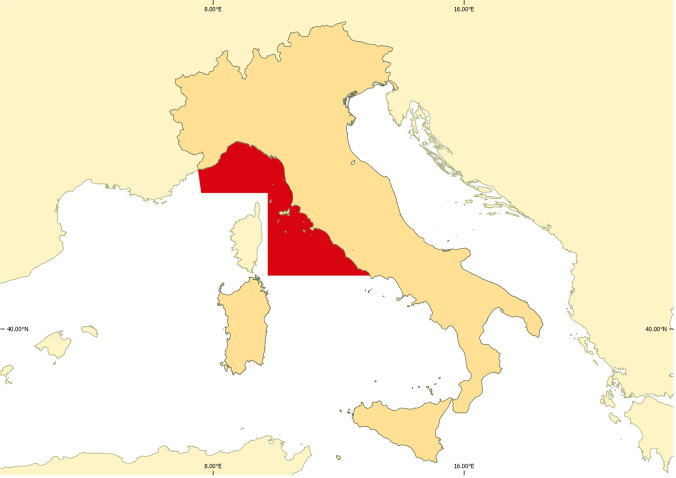
Map of the study area in the Ligurian and northern and central Tyrrhenian Seas (FAO-GFCM GSA9)

Atlantic horse mackerel specimens were sampled in July 2019 in the Ligurian and central-northern Tyrrhenian Seas (FAO-GFCM Geographic Sub-area 9) (Fig. 1) by trawling at depths ranging from 18 to 330 m during the implementation of the EU-funded Mediterranean international trawl survey (MEDITS project, Spedicato et al. 2019). After capture, samples were frozen immediately on board and transported to the Centro Interuniversitario di Biologia Marina “A. Bacci” (CIBM) labs for the analysis.

### Fish samples and parasitological examination

For each specimen, total length (TL, to 0.5 cm below) from the tip of the snout to the end of the tail and total weight (TW, g) (weighing scale precision 0.1 g) were recorded. Sex was determined through macroscopical examination of gonads.

Length–weight relationship was analyzed by means of the power equation W = aTL^b^, where W is the total weight and TL is the total length. The Le Cren (1951) relative condition factor (Kn), expressing the condition of a fish in numerical terms, was calculated from the observed total weight and theoretical weight (EW, g) estimated from “a” and “b” parameters of the length–weight relation.

For the parasitological examination, the abdominal cavity was examined by visual inspection, while the internal organs were observed under a stereomicroscope (magnification 8– × 35) for the presence of third-stage larvae (L3) of Anisakid and Raphidascaridid nematodes.

### Morphological analyses

All collected larvae were identified at the genus level according to their general morphology (Hartwich 2009; Gibbons 2010), through observation under light microscopy. The prevalence, mean intensity (MI), and mean abundance (MA) values of larvae belonging to each genus were calculated according to Bush et al. (1997).

### Molecular analyses

Genomic DNA was extracted from the central part of the larvae body by the PureLink® Genomic DNA Kit (Life Technologies, Carlsbad, CA) following the manufacturer’s instructions. Amplification of the complete ITS rDNA region was performed with primers NC5_f (5′-GTAGGTGAACCTGCGGAAGGATCATT-3′) and NC2_r (5′-TTAGTTTCTTCCTCCGCT-3′) (Zhu et al. 1998). The PCR products were electrophoresed on 1% agarose gel stained with SYBR Safe DNA Gel Stain (Thermo Fisher Scientific, Carlsbad, CA) in 0.5X TBE. For the polymerase chain reaction-restriction fragment length polymorphism (PCR–RFLP), 10 µl of the PCR product were digested with 1.5 µl of restriction enzymes *Hinf*I, *Hae*III, and *Alu*I (D’Amelio et al. 2000; Tedesco et al. 2018), in a volume of 20 µl at 37 °C for 90 min (Abollo et al. 2003). The restriction fragments were separated in 3% agarose gel stained with SYBR Safe DNA Gel Stain in 0.5X TBE. Sequenced *A. pegreffii* and *A. simplex* (s.s.) were used as positive controls in every reaction. After the electrophoresis, some specimens showed hybrid restriction patterns; therefore, in order to exclude the possibility of incomplete digestion, they were digested for longer time (240 min).

Sequenced *A. pegreffii* and *A. simplex* (s.s.) were used as positive controls (K +) in every reaction.

### Data analysis

The Chi-square test (significance level 0.05) was performed to assess possible significant differences in the prevalence of Anisakid and Raphidascaridid parasites between male and female fish and also to test the relationship between the prevalence of nematode parasites and depth.

The analysis was performed using the JMP statistical package (SAS, Jmp 2007).

Regarding the length–weight relationship, Student’s t-test was applied to test allometric growth (“b” = 3) (Pauly 1984) and differences between sexes.

Data exploration was performed to check correlation among variables (TL, TW, sex, Kn, and number of parasites), and a graphic output was produced (pairplot); the relationship between the number of parasites and biological parameters (TL, TW, sex) was tested by ANOVA. The possible effect of parasites on the condition factor was evaluated by Student’s t-test.

The prevalence of single or multiple infections of nematodes larvae with a 95% confidence level, based on the results of microscopic analysis, was calculated for the whole sample. The prevalence of Anisakids and Raphidascaridids in both fish sexes was subjected to statistical analysis, using the Chi-square test, and was considered significant at P < 0.05.

## Results

A total of 190 specimens of *T. trachurus* were collected during the MEDITS survey in July 2019, of which 107 were female and 83 were male. Body size ranged from 10.0 to 31.0 cm TL in females and from 10.0 to 31.5 cm TL in males.

Parasites were found in 70 individuals: 30 males and 40 females; prevalence, mean intensity, and mean abundance of parasites recorded for all specimens are reported in Table 1.

**Table 1:** Number of fish (*NF*), number of parasitized fish (*NPF*), prevalence (%), *CI* 95% confidence interval, range of intensity (*I*), min–max (average) (*RI*), abundance (*A*), number of parasites (*NP*)

	NF	NFP	%	CI	RI	A	NP
Males	83	30	36.14	29.20–43.09	1–6 (2.2)	1.12	93
Females	107	40	37.38	30.39–44.37	1–8 (2.36)	0.63	68
Total	190	70	37.89	29.87–43.81	1–8 (2.29)	0.85	161

A total of 161 visible larvae were collected in the viscera. Morphological analysis revealed the presence of 129 (28%) *Anisakis* spp. larvae in 55 fish: 21 males and 34 females and 31 larvae (10.5%) *Hysterothylacium* spp. in 15 fish: 9 males and 6 females, while 5 fish showed coinfection with both. All the values are reported in Table 2.

**Table 2:** Number of fish parasitized by *Anisakis* (*FPA*), number of fish parasitized by *Hysterothylacium* (*FPH*), prevalence (%), *CI* 95% confidence intervals, range of intensity (*RI*), min–max (average), abundance (*A*), number of *Anisakis* (*NA*), number of *Hysterothylaciu*m (*NH*). P = 0.032

FPA	%	CI	RI/min-max	A	NA	FPH	%	CI	RI/min–max	A	NH
21	25.30	19.02–31.58	1–6 (2.47)	0.57	47	9	10.84	2.22–19.46	1–4 (2.33)	0.253	21
34	31.77	25.05–38.50	1–8 (2.24)	0.77	83	6	5.61	1.64–9.58	1–3 (1.67)	0.0935	10
55	28.95	22.39–35.50	1–8 (2.35)	0.68	130	15	7.89	3.24–12.55	1–4 (2.07)	0.1632	31

Statistical analysis showed that the fish were more significantly infected with *Anisakis* larvae than with *Hysterothylacium* spp. (p = 0.032); however, no statistically significant differences in infection values were observed between sexes.

**Fig. 2:**
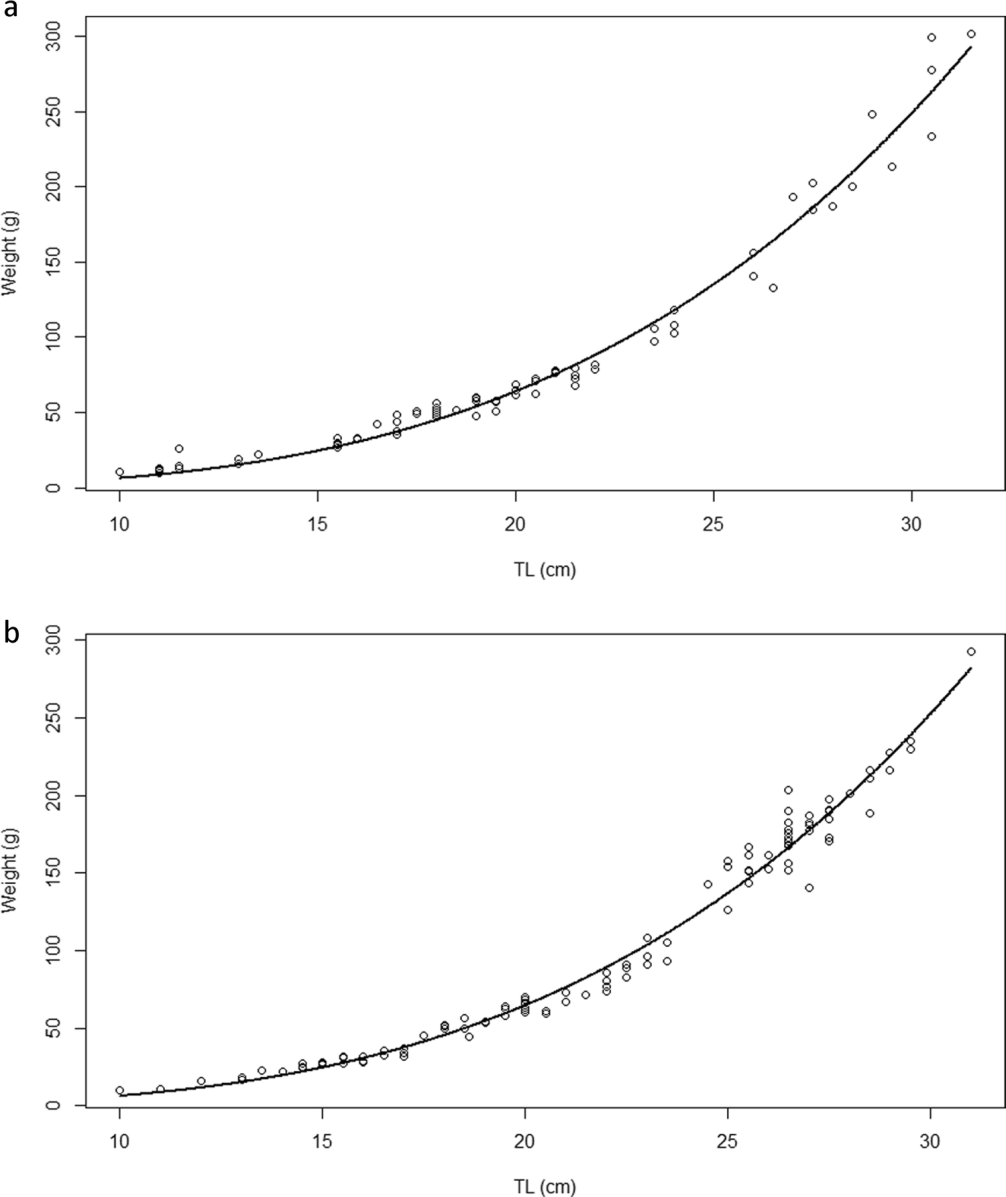
**a** Length–weight relationship in males of *Trachurus trachurus*. **b** Length–weight relationship in females of *Trachurus trachurus*

Length–weight relationship was calculated by sex and the results are shown in Fig. 2a and b  and Table 3. The “b” parameter differed significantly for each sex: females showed positive allometric growth, while males showed isometric growth. Statistically significant differences between sexes were not detected (t-value 0.498; p > 0.05).

**Table 3:** Length–weight relationship parameters for males and females of *Trachurus trachurus*. a and b are the parameters of the power function; SE(b), the standard error of b; r^2^, the coefficient of determination; and t-value, the value of the t-test

	a	b	SE(b)	r^2^	t-value
Males	0.0102	2.937	0.052	0.977	56.35
Females	0.0053	3.145	0.038	0.985	83.28

The condition factor (Kn) ranged from 0.80 to 1.98: the minimum values were 0.81 and 0.80 for males and females, respectively, while the maximum values were 1.98 for males and 1.27 for females. No statistically significant differences in the condition factor emerged between males and females (t = 0.190; p > 0.05) and between parasitized and non-parasitized individuals (t = 0.986; p = 0.325). Variations in the condition factor in relation to total length are shown in Table 3.

A preliminary data exploration highlighted a relationship between total length and total weight with the number of parasites (Pearson correlation coefficient (PCC) = 0.5); there was also a correlation between total length and total weight (PCC = 0.9).

A significant and positive correlation was found between the number of parasites and total length (t = 7.532; p < 0.05) and total weight (t = 8.786; p < 0.05), while sex was not significantly correlated (t = 0.925; p > 0.05).

The prevalence of nematode parasites was significantly higher (P < 0.0001) in horse mackerels caught at depths below 250 m (47.6%) compared to those captured above 250 m (23.5%). All (100%) the parasitized fish from deeper waters (> 250 m) were infected with *Anisakis* spp., while only one fish (1.9%) showed coinfection with *Hysterothylacium* spp. In contrast, the parasitized fish from shallower waters (< 250 m) were more frequently infected with *Hysterothylacium* spp. (70%) and less by *Anisakis* spp. (25%).

**Fig. 3:**
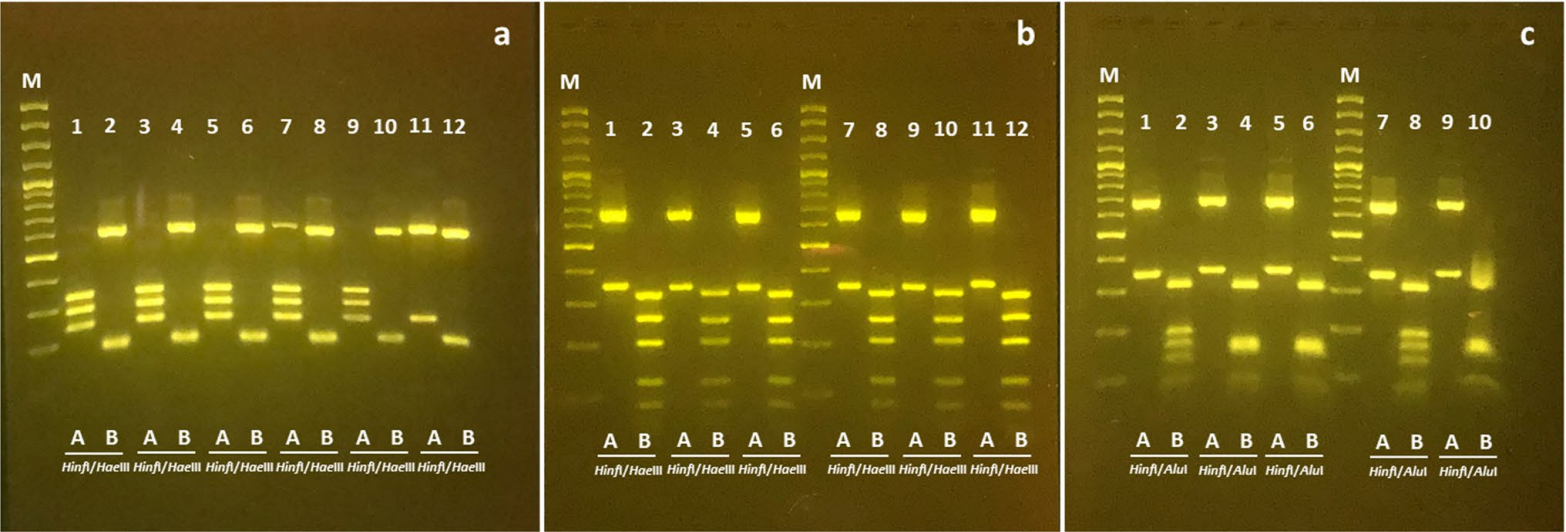
Restriction fragment length polymorphism patterns (molecular weight marker 100 base pairs) obtained with **a** restriction enzymes HinfI (lanes 1, 3, 5, 7, 9, and 11) and HaeIII (lanes 2, 4, 6, 8, 10, and 12), lanes 1–6 *Anisakis pegreffii*, lanes 7–8 *Anisakis pegreffii/Anisakis simplex* hybrid, lanes 9–10 positive control (K +) = *A. pegreffii*, lanes 11–12 K +  = *Anisakis simplex*; **b** restriction enzymes HinfI (lanes 1, 3, 5, 7, 9, and 11) and HaeIII (lanes 2, 4, 6, 8, 10, and 12), lanes 9–10 K +  = *Hysterothylacium aduncum*, lanes 11–12 K + *H. fabri*; and **c** restriction enzymes HinfI (lanes 1, 3, 5, 7, and 9) and AluI (lanes 2, 4, 6, 8, and 10), lanes 1–2 *H. aduncum*, lanes 3–6 *H. fabri*, lanes 7–8 K +  = *H. aduncum*, lanes 9–10 K +  = *H. fabri*

With regard to molecular analyses, all the specimens subjected to PCR (n = 67) were successfully amplified, showing bands of ~ 1000 bp. The PCR–RFLP showed that 85% (n = 47) of the *Anisakis* larvae analyzed belonged to the species *A. pegreffii*, while in the remaining 15% (n = 8), hybrids of *A. pegreffii*-*A. simplex* (s.s.) were detected (Fig. 3a).

A total of 58% (n = 7) of the *Hysterothylacium* larvae analyzed belonged to the species *H. fabri*, while 42% (n = 5) belonged to the species *H. aduncum* (Fig. 3b and c). For confirmation, the hybrids were re-digested with the same enzymes for 240 min.

## Discussion

The present study provides information on the distribution of third-stage larvae of *A pegreffii*, *H. aduncum*, and *H. fabri* in *T. trachurus* from the Ligurian and Tyrrhenian Seas (western Mediterranean), correlating the infection data with the biological and biometric features of the hosts.

Our results highlighted that the genus *Anisakis* (28.95%) was more prevalent than *Hysterothylacium* (7.89%). This coinfection pattern is in accordance with other parasitological investigations on *T. trachurus* from the Mediterranean and Extra-Mediterranean regions. Fioravanti et al. (2003) reported a higher prevalence of *Anisakis* (33.7%) compared to *Hysterothylacium* (12.2%) in *T. trachurus* from the central Adriatic Sea. With respect to the Ligurian Sea, in horse mackerels Serracca et al. (2013) reported a prevalence of 15.6% for *Anisakis* and 9.3% for *Hysterothylacium* larvae. Manfredi et al. (2000) reported higher prevalence values (80–100%) only for *Anisakis* spp. In a survey carried out on *Trachurus* spp. caught off the coast of Sardinia, Angelucci et al. (2011) reported prevalences of 52.5% for *Anisakis* spp. and 77.9% for *Hysterothylacium* spp. In *T. trachurus* fished off the coasts of Sicily, Costa et al. (2016) found a 6.7% prevalence for *H. aduncum*. Goffredo et al. (2019) reported prevalence values of 50.8% for *Anisakis* and 0.54% for *Hysterothylacium* in *T. trachurus* from the Ionian Sea. MacKenzie et al. (2008) analyzed the parasite fauna of *T. trachurus* in different sampling stations across the northeastern Atlantic and Mediterranean Seas, reporting that *Anisakis* spp. and *H. aduncum* were the most common parasites detected in horse mackerel. Their results also highlighted the usefulness of *Anisakis* spp. and *Hysterothylacium* spp. as biological tags for distinguishing different horse mackerel stocks and identifying migration patterns.

The high prevalence of Anisakis is probably also related to the common practice of local fishermen, who discard the fish viscera directly at sea. These viscera then become a food source for a variety of fish, cetaceans, and seabirds, which can thus ingest any larvae of *Anisakis* that may be present (Oro and Ruiz 1997; Morton and Yuen 2000; Arcos et al. 2001; Bozzano and Sardà 2002). In the Ligurian Sea, it is also possible that the high prevalence of *Anisakis* is linked to the presence of the “Pelagos Sanctuary,” a marine protected area with a high density of marine mammals which are definitive hosts of this genus (Mattiucci et al. 2004; Mattiucci and Nascetti 2006, 2008).

Concerning the molecular analysis, the PCR–RFLP identified *A. pegreffii* and hybrids *A pegreffii*-*A. simplex* and *H. fabri* and *H. aduncum*. The hybrid *A. pegreffii*-*A. simplex* (s.s.) has been described in *T. trachurus* from the Cantabrian Sea (Abollo et al. 2003) and from the coasts off Sardinia (Meloni et al. 2011).

The results are in agreement with the evidence that *A. pegreffii* is the dominant *Anisakis* species in the Mediterranean Sea, as highlighted by Mattiucci et al. (2018). The occurrence of hybrids in the Mediterranean Sea, detected through PCR–RFLP of the ITS region of rDNA and other molecular markers, has also been reported by numerous studies in other fish species and in marine mammals (Abollo et al. 2003; Meloni et al. 2011; Cavallero et al. 2012; 2014).

The reason for the spread of hybrid genotypes in the Mediterranean is still unclear (Meloni et al. 2011). In the northeastern Atlantic and in the western Mediterranean, *A. simplex* (s.s.) and *A. pegreffii* are known to occur in sympatry (Abollo et al. 2001; 2003) and may undergo interspecific hybridization. However it is unclear whether this phenomenon results in a higher or lower fitness of hybrids compared to parental species and therefore in a higher or lower infectivity or the possibility of parasitizing different host species. Future investigations considering multiple molecular markers (Mattiucci et al. 2018) may shed further light on these aspects.

With respect to the biological and biometric features of the *T. trachurus* examined, the “b” values for females (b = 3.145) showed positive allometric growth, with the growth in length proportionally bigger than the growth in weight. For males (b = 2.937) and the total sample, isometric growth was recorded. Similar results have been found in other areas of the western (Gancitano et al. 2011; Ligas et al. 2012; Spedicato et al. 2012) and eastern (Lembo et al. 2012; Carbonara et al. 2012; Santojanni et al. 2013) Italian Seas.

Le Cren’s condition factor was applied to assess the fish welfare linked with the length–weight relationship, which can be influenced by parasites (Dias et al. 2015; Silva et al. 2013; Santos et al. 2013) as well as factors such as gonad maturation and feeding (Verani et al. 1997).

A strong correlation (P < 0.0001) was found between the number of nematode parasites in the viscera and the body size. However, our results suggest that Anisakid and Raphidascaridid parasites do not influence the state of health of the horse mackerel in terms of body condition. This result is in accordance with the results of a previous study (Ichalal et al. 2015) which failed to detect a negative impact of *A. simplex* and *H. aduncum* on the condition of *T. trachurus* based on the analysis of Fulton’s condition index. In fact, very few studies have explored the effect of *Anisakis* infection on the body condition of fish and with contrasting results (Podolska and Horbowy 2003; Lagrue and Poulin 2015).

In our study, a trend in Kn value was observed in relation to the length, but it did not differ from 1. Kn increased between 16.5 and 18.0 cm TL and then decreased. A similar trend was recorded by Alegria-Hernandez (1994) and Šantić et al. (2011), which is linked to the development and maturation of the gonads: after length at first maturity, 18.8 cm in GSA9, (MEDISEH 2013), Kn values decrease due to the high energy demand required by reproduction. Differences in the maturity stage of the fish could therefore mask the effect of parasitic infections on the body condition of the fish host and result in the contrasting evidence found in the literature.

With respect to body size, we found a positive correlation between fish size and the prevalence of *Anisakis*, in accordance with the results of several parasitological surveys on different fish species (Mattiucci et al. 2018 and references therein), suggesting that fish size could be a good predictor of infection with *Anisakis* spp. and of the associated risk of anisakiasis in humans (Madrid et al. 2016). However, other research failed to detect any relationship between fish length and the number of *Anisakis* larvae in the edible parts of fish (Karl et al. 2011).

In addition, the time after capture and storage temperature can play an important role in defining the distribution of *Anisakis* larvae in fish filets (Cipriani et al. 2016). The relationship between fish size and the zoonotic potential of *Anisakis* in the fish host is therefore not always obvious.

Fish age, which is positively correlated to body size, is one of the main factors to be considered in the analysis of infection levels in long-lived parasites, such as *Anisakis* spp. (Abaunza et al. 1995). Higher infection levels in older and larger fish are the result of a bioaccumulation of parasites throughout the fish’s life span and, possibly, of ontogenetic dietary shifts. Furthermore, larger fish feed at a higher rate with a variety of potential intermediate/paratenic hosts, thus favoring higher parasitization levels (Abattouy et al. 2011). In fact, larger fish tend to occupy higher levels in the food chain with the increased possibility of ingesting intermediate/paratenic hosts parasitized with Anisakids (Strømnes and Andersen 2000).

In the present study, the sampled fish were stratified according to the depth of capture (< 250 m and > 250 m). Our results showed a statistically significant correlation values (P < 0.0001) between depth and prevalence of nematode parasites, which are more prevalent in fish from deeper waters (> 250 m). Such a correlation could be explained by the presence of larger (thus more parasitized) fish at a greater depth. However, this variable appears to be a main risk factor for Anisakid and Raphidascaridid infection in commercially important marine fish, as previously reported in a variety of teleost species (e.g., *Sardina pilchardus*, *Engraulis encrasicolus*, *Phycis blennoides*) independently of fish size (Pulleiro-Potel et al. 2015). Specific oceanographic and ecological factors, such as temperature, oceanic currents, depth, salinity, and primary production, have been identified as the main variables affecting the distribution of *Anisakis* spp. (Højgaard 1998; Kuhn et al. 2016).

## Conclusion

In conclusion, our study provides information on the infection pattern of *Anisakis* and *Hysterothylacium* larvae in *T. trachurus* from the Ligurian and Tyrrhenian Seas, and the occurrence of the species *A. pegreffii* together with *A. pegreffii*/*A. simplex* (s.s.) hybrids, and *H. aduncum* and *H. fabri*, identified by molecular methods. Our results also support the hypothesis that infection with these parasites does not affect the condition of the fish host analyzed, and that body size and depth are major drivers in determining infection levels with Anisakid and Raphidascaridid nematodes.

## Data Availability

Not applicable.
